# Use of infographics for facilitating learning of pharmacology in the nursing degree

**DOI:** 10.1002/nop2.1413

**Published:** 2022-10-20

**Authors:** Alfonso Meneses‐Monroy, Ana B. Rivas‐Paterna, Elena Orgaz‐Rivas, Francisco J. García‐González, María J. González‐Sanavia, Guillermo Moreno, Enrique Pacheco

**Affiliations:** ^1^ Nursing Department, Faculty of Nursing, Physiotherapy and Podiatry Universidad Complutense de Madrid (Complutense University of Madrid) Madrid Spain; ^2^ Research institute imas12 Hospital Universitario 12 de Octubre (University Hospital 12 de Octubre) Madrid Spain; ^3^ Processes, Research, Innovation and Information Systems Unit, Directorate of Nursing Instituto de Investigación Sanitaria San Carlos (San Carlos Health Research Institute ‐IDISSC), Hospital Clínico San Carlos (San Carlos Clinical Hospital) Madrid Spain

**Keywords:** infographics, nursing degree, teaching methodology

## Abstract

**Aim:**

To evaluate the impact of an educational intervention focused on teaching students to create infographics to improve pharmacology learning.

**Design:**

This is a comparative study.

**Methods:**

The population was 250 nursing students who had to create two infographics in groups related to the content that had been addressed in pharmacology in two different moments. Students and professors evaluated the infographics through a 5‐point Likert scale. Scores from the official exam of the pharmacology subject were obtained.

**Results:**

Most of the students scored below 50% for the “excellent” and “good” categories. Intraclass correlation and kappa correlations among students and professors' evaluations were low. The comparison between both times of students' evaluations only yields significant correlation values for the criterion “Understanding of information” (*r* = .039, *p* = .024) and the “Visual presentation of information” (*r* = .041, *p* = .019). No correlation was obtained between the test and evaluations values of the infographic.

## 1 INTRODUCTION

Pharmacological knowledge is fundamental for professional nurses to avoid errors while delivering care. However, many students still perceive it as a complex subject (Khan & Hood, 2018). Because of this, it's always been interesting to find instruments which might facilitate learning on this subject, whether it's through new learning methods or new technologies (Mauldin, 2021; Sajjad & Gowani, 2021; Tinnon & Newton, 2017).

## 2 BACKGROUND

As new information and communication technologies (ICT) are incorporated into higher education, new tools are becoming available and helping to achieve a student's knowledge. In this context, infographics have been implemented in higher education institutes (Kocsis Baan et al., 2018). Infographics are a combination of “information” and “graphics”. The infographics have a design where the data is combined with images to facilitate the dissemination of information (Noh et al., 2015). In recent years, infographics are presented as a teaching tool to help the instructors in the teaching and learning session to facilitate the students (Noh et al., 2015). With the use of the Internet and social media, infographics have become a popular format to share medical information all over the world. They allow for turning complex information into a visual, attractive, didactic and shareable format (Hernandez‐Sanchez et al., 2020).

According to some authors, infographics are appropriate for any student's level and different learning environments, such as classrooms, clinical settings or online (Chicca & Chunta, 2020).The use of infographics to produce a healthcare information has proved to be useful in different situations in the education system. For instance, Tew et al. developed key information aimed at intermittent claudication patients and got better results and better benefits by using infographics (Tew et al., 2020). Provvidenza et al. used infographics to teach students about concussions, and students identified them as having met their knowledge needs (91%) and provided new knowledge (87%). Students also declared having the intention of using infographics to develop their knowledge (89%) and teach others (55%) (Provvidenza et al., 2019). Cima et al. developed a training system through infographics that was distributed through social networks with medicine students, improving their results in the acquisition of Anatomic Pathology knowledge (Cima et al., 2021). Shanks et al. teach undergraduate students to communicate public health data with infographics. For the students, it was clear that the infographic task facilitated learning about public health and the overall experience was received as positive (Shanks et al., 2017). Besides, over undergraduate students, infographics has a favourable impact on academic accomplishment, visual thinking skills, willingness to learn (Samra, 2021) and allow undergraduate students to delve into the study topics of a subject (Malik & Nakhla, 2020). Despite the multiple possibilities and uses of infographics in different educational contexts, there are no studies that have specifically assessed the usefulness of infographics to facilitate learning of pharmacology as a subject for the Nursing Degree. Therefore, the aim of the study is to evaluate the impact of an educational intervention focused on teaching students to create infographics to improve pharmacology learning.

## 3 METHODS

### 3.1 Setting and population

This study was carried out in 2019 at a Spanish university. The population was 250 second‐year nursing students enrolled in the subject of Pharmacology. The subject is taught in a format of master class and seminars, with a final exam. The number of professors of the subject was seven.

### 3.2 Design and intervention

The study has a comparative design with a single sample of the total number of students and consists of the evaluation of an educational intervention in which the students had to carry out in groups two infographics related to the contents that had been addressed in the pharmacology subject in two different times when the data was obtained.

The first part of the intervention consisted in the design of an infographic as a task of the subject where the students were only informed of the rules for carrying it out. Regarding the first infographic, its norms were published through the virtual campus, in which groups of five students were randomly organized. In the same way, a topic was randomly assigned from those addressed up to that point in the course (autonomic nervous system drugs). All students participated and created an infographic.

After completing the assignment, the second part of the intervention consisted of an online intervention by the teachers of the subject in which online support material about the design of infographics was uploaded. The written information was published on the virtual campus (e‐learning application), so that all students had access. These materials focused on the structure and characteristics of infographics, with the aim that students could answer two questions that would allow them to improve their design skills:

- *What is an infographic?* We provide them written information, and web links where characteristics of the infographics were explained (the size and dimensions they have, the definition of infographics, the combination of text and graphics or figures that characterizes them, their informative purpose, etc.) Besides, examples of infographics were uploaded.
- *What structure should an infographic have?* In the same way, written information and information sources were provided through the Internet, where it was explained how to organize the elements of an infographic, the amount of data that should appear, the number of words, etc.

Likewise, there was created a forum on the virtual campus where students could raise any doubts about the designs of infographics, and they were all answered by the professors of the subject.

Subsequently, the last and third part of the intervention consisted in the realization of a second infographic by the students, following the same criteria defined in the first infographic but related to a different topic of the subject, selected randomly (antibiotics). The time between both infographics was approximately one month.

On the one hand, it was hypothesized that the design of infographics by the students themselves favoured their autonomous work and acquisition of knowledge and, on the other hand, through the professors' materials and assistance, the skills of creating infographics were improved.

### 3.3 Variables

To evaluate the effect of the intervention in facilitating learning of the topics of the pharmacology subject addressed in the infographics, scores of these topics (autonomic nervous system drugs and antibiotics) from the official exam of the pharmacology subject were obtained. Besides, immediately after making the infographics, all students had to evaluate the acquisition of knowledge of pharmacology of their peers through evaluation of all their peers' infographics, in both times; the professors of the subject (as objective evaluators) also evaluated students' learning by evaluating all the infographics to verify that the students were able to objectively evaluate their peers. To carry out the evaluation of their peers, the students were instructed (through an instruction document that was uploaded to the virtual campus) how to evaluate the infographics. The evaluation criteria were the same and through an online form that students and professors had access to, with five criteria: “Visual presentation of the information”, “Scientific quality of the information presented”, “Understanding of the information”, “Adequacy of the information presented to the proposed topic” and “Bibliography used”. Each criterion was evaluated using a 5‐point Likert scale: excellent, good, acceptable, not very acceptable and unacceptable. Each level of the scale was assigned a numerical value (Table 1).

**TABLE 1:** Scoring evaluation of the infographics using a Likert scale and its conversion into numerical values

Criterion	Excellent	Good	Acceptable	Not very acceptable	Unacceptable
Visual presentation of information	1 point	0.75 points	0.5 puntos	0.25 points	0 points
Scientific quality of information	1 point	0.75 points	0.5 puntos	0.25 points	Or points
Understanding of information	1 point	0.75 points	0.5 puntos	0.25 points	0 points
Adequacy of the information presented to the proposed topic	1 point	0.75 points	0.5 puntos	0.25 points	0 points
Bibliography used	1 point	0.75 points	0.5 puntos	0.25 points	0 points

### 3.4 Statistical analysis

Scores from the topics of the exam are presented as means and standard deviations. Evaluation of both infographics by using the Likert scale are presented as percentages (but analyses were also performed converting them into numerical scores as seen in Table 1). An analysis of the Kappa coefficient and the intraclass correlation was carried out to identify the uniformity of the evaluation between the students and the professors. Pearson and Spearman correlations analysis were performed to assess the relationships between the students and the professors' evaluations. Kendall's tau was performed with the correlations to analyse the differences related to the sample size (the number of professors' evaluations was low in relation to the students) and the differences that could be present when converting the variables from qualitative to quantitative. Differences between both times of evaluation were compared by chi‐square when appropriate for categorical variables and by paired Student's *t*‐test for numerical variables (we have compared the numerical means of both evaluations of the students and the professors) to check for improvements over time. Significance was defined for a 95% confidence interval and a *p*‐value <.05.

### 3.5 Ethics

Ethical approval was obtained from the academic board of our faculty before the start of the course. All the students were invited to participate in the study, and all were informed of the objectives and terms of implementation of the research at the start of the course and again later after the beginning of the activity. Oral consent was obtained from all participants before the start of the activity. Besides, if any student refused to participate, an alternative evaluation by clinical case was provided.

The principles enshrined in the Helsinki Declaration on Biomedical Research Involving Human Subjects were observed at all times. The confidentiality and privacy of their information were observed in compliance with current regulations on the protection of personal data. The data were entered in secure databases and access to the data was restricted to the researchers. Data analysis was limited to the purposes of this study.

## 4 RESULTS

The number of students participating in the study was 250. The total number of evaluations was 3,241, from 64 to 65 for each infographic.

Regarding the percentages of the students' Likert evaluations, most of them scored below 50% for the “excellent” and “good” categories (Table 2). Besides, there were also only major differences in visual presentation (*p* = .011) and understanding of information (*p* = .005) between evaluations (Table 2).

**TABLE 2:** Students' evaluations of the Likert scale criteria of the infographics (*n* = 250)

Students		Visual presentation (%)		Scientific Quality (%)		Understanding of information (%)		Adequacy of information (%)		Bibliography used (%)	
		Infographic 1	Infographic 2	Infographic 1	Infographic 2	Infographic 1	Infographic 2	Infographic 1	Infographic 2	Infographic 1	Infographic 2
Unacceptable	0	0.31%	0.50%	0.06%	0.06%	0.09%	0.23%	0.18%	0.26%	2.03%	0.09%
Not very acceptable	2.5	6.21%	5.82%	1.38%	1.78%	2.24%	2.28%	2.43%	2.46%	1.84%	1.61%
Acceptable	5	21.46%	24.76%	11.65%	13.91%	14.97%	17.33%	12.73%	15.23%	9.62%	11.05%
Good	7.5	34.49%	36.28%	48.14%	46.54%	39.59%	42.24%	38.46%	40.51%	36.12%	36.83%
Excellent	10	37.53%	32.65%	38.76%	37.71%	43.10%	37.91%	46.20%	41.54%	50.38%	50.42%
		Chi square	*p*‐value	Chi square	*p*‐value	Chi squared	*p*‐value	Chi square	*p*‐value	Chi square	*p*‐value
		37,114	.011	21,970	.327	39,870	.005	26,699	.144	14,484	.805

*Note*: Data presented as percentage of students' answers to each criterion.

In the same way, the evaluations of the professors in each of the evaluation criteria is presented in Table 3, where no significant differences where observed, and most of the scores were below 50% in the “excellent” and “good” categories.

**TABLE 3:** Professors' evaluations of the Likert scale criteria of the infographics (*n* = 7)

Professors		Visual presentation (%)		Scientific quality (%)		Understanding of information (%)		Adequacy of information (%)		Bibliography used (%)	
		Infographic 1	Infographic 2	Infographic 1	Infographic 2	Infographic 1	Infographic 2	Infographic 1	Infographic 2	Infographic 1	Infographic 2
Unacceptable	0	0	0	0	0	0	0	0	0	4.30%	0
Not very acceptable	2.5	17.40%	34.80%	21.70%	0	17.40%	0	13%	0	17.40%	34.80%
Acceptable	5	39.10%	52.20%	30.40%	39.10%	26.10%	34.80%	26.10%	4.30%	47.80%	43.50%
Good	7.5	43.50%	13%	43.50%	47.80%	43.50%	52.20%	52.20%	30.40%	26.10%	21.70%
Excellent	10	0	0	4.30%	13%	13%	13%	8.70%	52.20%	4.30%	0
		Chi square	*p*‐value	Chi square	*p*‐value	Chi square	*p*‐value	Chi square	*p*‐value	Chi square	*p*‐value
		1,576	.813	5,118	.529	3,274	.774	4,883	.844	3,942	.862

*Note*: Data presented as percentage of professors' answers to each criterion.

The comparison of students and professors' evaluations in each of the study's times did not show significant results, except for Kendall's Tau in “Scientific Quality of Information” (*r* = .296, *p* = .016) as presented in Table 4.

**TABLE 4:** Comparison between students (*n* = 250) and professors (*n* = 7) evaluations of each of the criteria of the Likert scale for the first and second infographic

	Infographic 1		Infographic 2	
	Student/Professor ratio		Student/Professor ratio	
		*p*‐value		*p*‐value
Visual presentation of information				
Kendall's Tau	−0.009	.893	−0.135	.478
Spearman C.	−0.01	.894	−0.151	.492
Pearson C.	−0.16	.828	−0.147	.502
Scientific quality of information				
Kendall's Tau	0	.995	0.296	.016
Spearman C.	−0.001	.991	0.338	.114
Pearson C.	−0.001	.987	0.322	.135
Understanding of information				
Kendall's Tau	−0.001	.986	0.167	.972
Spearman C.	−0.001	.986	0.007	.974
Pearson C.	0.037	.618	0.059	.788
Adequacy of the information presented				
Kendall's Tau	0.01	.875	0.024	.897
Spearman C.	0.011	.881	0.024	.915
Pearson C.	0.007	.927	0.004	.987
Bibliography used				
Kendall's Tau	0.034	.616	0.24	.204
Spearman C.	0.039	.601	0.261	.228
Pearson C.	0.047	.524	0.229	.294

Intraclass correlation and kappa correlations among students and professors' evaluations were low, except for “Understanding of information” (*p* < .001) (Table 5).

**TABLE 5:** Data on the correlation value of the kappa coefficient and the intraclass correlation and its level of significance between students' (*n* = 250) and professors' evaluations (*n* = 7)

Infographic 1/Inphografic 2			Infographic 1		Inforgraphic 2	
	Kappa	Significance	Intraclass correlation	Significance	Intraclass correlation	Significance
Visual presentation of information	−0.007	.535				
Scientific quality of information	0.005	.713				
Understanding of information	0.017	.164	0.417	<.001	0.53	<.001
Adequacy of the information presented	−0.003	.818				
Bibliography used	0.001	.912				

The comparison between both times of students' evaluations only yields significant correlation values for the criterion “Understanding of information” (*r* = .039, *p* = .024) and the “Visual presentation of information” (*r* = .041, *p* = .019). However, statistical differences were found in every criterion when mean scores of students' evaluations between infographics were compared. It is observed that the value of the Bibliography used is negative so that the Bibliography used in the pre‐intervention is higher than the postintervention; both values have a significance of less than *p* < .05 (Table 6). When comparing scores between the evaluations of professors, separated in the two periods, no significance values of *p* < .05 were obtained in any of the criteria evaluated.

**TABLE 6:** Calculation of Student's *t* of the Likert scale evaluation criteria between first and second infographics in students (*n* = 250)

	Infographic 1/Inphographic 2 students			
	Correlation	*p*‐value	t	*p*‐value
Visual presentation of information	0.041	.019	3.394	<.001
Scientific quality of information	0.017	.324	2.679	.007
Understanding of information	0.039	.024	4.621	<.001
Adequacy of the information presented	0.005	.759	4.127	<.001
Bibliography used	−0.026	.135	−2.443	.015

Also calculated were the average scores of the official exam of the topics addressed by the infographics obtained by the group of students and the average score of the evaluations of the infographics. Thus, of the topics addressed in the first infographic, the average score of the test was 5.65 SD: 1.54 and 8.08 SD: 1.39 of the infographics. Of the topics addressed in the second infographic, a mean test score of 5.47 SD: 1.62 was obtained, and infographic values of 7.79 SD: 1.73. No correlation was obtained between the test and evaluations values of the infographic, with a significance >.05.

## 5 DISCUSSION

One of the most relevant elements of the study was to verify whether using infographics expanded the student's knowledge and favours achieving the competencies needed for the subject of pharmacology. However, the evaluations on the Likert scale obtained by the students from their peers, were low, as from the professors, in both moments. Most evaluations scored below 50% for the “excellent” and “good” categories. Differences between times of evaluation of each criterion of the Likert scale provided by the students shows that there are no improvements in infographics scores over time in consensus with the professors scores. However, when numeric scores provided by the students are compared, it seems that there are improvements over time in every criterion, although there are no improvements in numeric scores provided by the professors. Correlations among students and professors were low and even the relationships were negative in some cases. Finally, scores from the official exam were lower than the scores obtained at each infographic and there were no significant correlations among them.

Although students' numerical scores improve over time, these data have not been corroborated when comparing the data of the students with the scores provided by the professors. Besides, the values of the correlation of the kappa coefficient and the intraclass correlation indicates there is no agreement, except for “Understanding of information”. The scores of this competency of the Likert scale are the most similar ones between evaluators which may be logical since the professors tend to undervalue the most technical aspects of the infographics (Table 3) such as the scientific quality of the information or the use of the bibliography while students tend to overvalue it (Table 2), so we thought that the understanding of the information in not a high technical competence, and that explains why the correlations are significant. The weak correlations among students and professors seems to indicate that there is no agreement between the evaluations of the professors and the students in the quality of the learning obtained. Professors' rate learning far below what students rate it. This could indicate that from the perspective of reference evaluators such as university professors, the use of infographics is not increasing student learning.

To sum up, according to the data obtained, it does not seem that these actions have translated to achieving competencies of the subject.

In contrast with our results, other authors found improvements in the acquisition of knowledge in a sample of clinicians who answered tests correctly 1.5 times more often after following infographics introductions (Hughes et al., 2021). Shanks, in a qualitative study with a sample of undergraduate health students, received positive feedback from them in the development of professional skills, communicating science‐related topics to non‐experts, and understanding health issues (Shanks et al., 2017), whereas other researchers, in a sample of undergraduate students using infographics to improve acquisition of radiology knowledge, conclude that there is no clear causal relationship between quantity of knowledge and the effect of lecture using infographics. Also, they deduce from their results that infographic educational materials on each topic had a different effect on knowledge levels, suggesting that more effective materials could be developed based on the different competencies and knowledges (Takashima et al., 2019). Vega‐Garzón used infographics and other complex forms of visual data analysis with medicine and veterinary students to teach parasitology. They found that after the intervention the grade value was higher and 93.75% of the students considered it a useful tool (Vega‐Garzón et al., 2022). In the same way, other research groups conducted a study of infographics in health education in Saudi Arabia and concluded that they were of high quality and recommended designing and making infographics on public health and using it through social networks (Jahan et al., 2021).

Regarding the importance given by students to the acquisition of knowledge through infographics, in a study made in a sample of non‐major science students, authors found that 13% of students explicitly referenced infographics in their reflections of acquisition (Adkins‐Jablonsky et al., 2021). Also, other researchers have created a series of six infographics which were given to a sample of teachers, students and healthcare professionals. 91% of the participants identified that the infographics met their knowledge needs and 87% recognize that the infographics provided them new knowledge (87%) (Provvidenza et al., 2019). Finally, another research group, in a similar study, using a sample of undergraduate Biochemistry and General Chemistry students, where they had to create infographics on topics related to the four major classes of biomolecules, found that students reported positive learning gains on Biochemistry concepts related to the four biomolecules. The students viewed favourably the creation of infographics, and they indicate that they have acquired digital competencies to effectively represent and visualize their understanding of biochemical concepts and explain these processes to peers (Sahai & Ivanova, 2022).

Since we do not have satisfaction measures of the students with the creation of infographics, it is difficult for us to compare our results to other authors. Besides, we have not found studies where samples of nursing students have been used or studies that use infographics to evaluate the acquisition of knowledge and competencies in pharmacology. On the one hand, the results provided by other authors allow us to trust the use of infographics in education. On the other hand, the lack of studies similar to ours (in pharmacology) means that this educational methodology is innovative and opens the possibility of creating new educational interventions based on the development of infographics to improve the acquisition of nursing knowledge and skills.

While managing technology, evidence and information by the target population group should have improved competencies, in our case, it seems that the students were not clear about the aim of the infographic, leading to data being used without analysing and incorporating it into their knowledge.

All of these data may be related to the infographic presentation so that the students could use their previous knowledge or information to develop them. Likewise, there is an implicit “wrongdoing” incorporated into the way of doing it. The infographics are related to the student's grade, which leads them to use any resource to obtain the desired favourable outcome.

### 5.1 Limitations

This is a single‐centre study, so our findings may not be generalizable to other centres or other health sciences disciplines since our sample was composed with second‐grade nursing students. Besides, the lack of a control group does not allow us to know if there is an improvement in the acquisition of knowledge and competencies in pharmacology with the use of infographics compared to traditional teaching methods. Future studies should be carried out with students from other disciplines, larger samples and an experimental design with a control group.

## 6 CONCLUSIONS

The structured infographics presented in the study do not facilitate learning of pharmacology of the students in the subject.

The intervention, structure, organization, and information should be improved and adapted to what has been defined by other authors since it may have generated confusion in the elements to be developed in the infographics.

It would be necessary to hold a workshop on the realization of infographics, using the elements that compose them and their characteristics, to establish the knowledge and content in the technique of infographics.

It would be wise that students better identify the criteria of the subject, which would help us identify the criteria that will be used, which correspond to the level of knowledge that the students should acquire in each of the topics and thematic blocks of the subject.

In any case, infographics as a teaching resource can be very interesting, introducing the changes above, since they incorporate in concrete actions (a topic, a pharmacological group) the entire structure of searching, deepening, identifying key elements and highlighting the evidence of each part.

## AUTHOR CONTRIBUTIONS

Made substantial contributions to conception and design, or acquisition of data, or analysis and interpretation of data: Alfonso Meneses‐Monroy, Enrique Pacheco; Involved in drafting the manuscript or revising it critically for important intellectual content: Alfonso Meneses‐Monroy, Enrique Pacheco; Given final approval of the version to be published: all authors. Each author should have participated sufficiently in the work to take public responsibility for appropriate portions of the content: Alfonso Meneses‐Monroy, Ana B. Rivas‐Paterna, Elena Orgaz‐Rivas, Francisco J. García‐González, María J. González‐Sanavia, Guillermo Moreno and Enrique Pacheco; Agreed to be accountable for all aspects of the work in ensuring that questions related to the accuracy or integrity of any part of the work are appropriately investigated and resolved: Alfonso Meneses‐Monroy, Ana B. Rivas‐Paterna, Elena Orgaz‐Rivas, Francisco J. García‐González, María J. González‐Sanavia, Guillermo Moreno and Enrique Pacheco.

## FUNDING INFORMATION

Guillermo Moreno is supported by a pre‐doctoral grant by the Spanish Ministry of Education (FPU18/03606).

## CONFLICT OF INTEREST

The authors declare that they have no conflict of interest in relation to the development of the research work presented. The research work was carried out after the approval of the Ethics and Research Committee of the Faculty of Nursing, Physiotherapy and Podiatry. The reference number of said approval is: FEFP 20/21. All the orientations manifested in the Declaration of Helsinki and later, as well as the legislation in force in our country, have been followed.

## Data Availability

Data available on request from the authors.
